# Novel Assessments of Technical and Nontechnical Cardiac Surgery Quality: Protocol for a Mixed Methods Study

**DOI:** 10.2196/22536

**Published:** 2021-01-08

**Authors:** Donald Likosky, Steven J Yule, Michael R Mathis, Roger D Dias, Jason J Corso, Min Zhang, Sarah L Krein, Matthew D Caldwell, Nathan Louis, Allison M Janda, Nirav J Shah, Francis D Pagani, Korana Stakich-Alpirez, Milisa M Manojlovich

**Affiliations:** 1 Department of Cardiac Surgery University of Michigan Ann Arbor, MI United States; 2 Department of Clinical Surgery University of Edinburgh Edinburgh United Kingdom; 3 Department of Anesthesiology University of Michigan Ann Arbor, MI United States; 4 STRATUS Center for Medical Simulation Department of Emergency Medicine, Brigham and Women's Hospital Harvard Medical School Boston, MA United States; 5 Department of Electrical Engineering and Computer Science School of Engineering University of Michigan Ann Arbor, MI United States; 6 Department of Biostatistics School of Public Health University of Michigan Ann Arbor, MI United States; 7 Department of Internal Medicine University of Michigan Ann Arbor, MI United States; 8 School of Nursing University of Michigan Ann Arbor, MI United States

**Keywords:** cardiac surgery, quality, protocol, study, coronary artery bypass grafting surgery, complications, patient risk, variation, intraoperative, improvement

## Abstract

**Background:**

Of the 150,000 patients annually undergoing coronary artery bypass grafting, 35% develop complications that increase mortality 5 fold and expenditure by 50%. Differences in patient risk and operative approach explain only 2% of hospital variations in some complications. The intraoperative phase remains understudied as a source of variation, despite its complexity and amenability to improvement.

**Objective:**

The objectives of this study are to (1) investigate the relationship between peer assessments of intraoperative technical skills and nontechnical practices with risk-adjusted complication rates and (2) evaluate the feasibility of using computer-based metrics to automate the assessment of important intraoperative technical skills and nontechnical practices.

**Methods:**

This multicenter study will use video recording, established peer assessment tools, electronic health record data, registry data, and a high-dimensional computer vision approach to (1) investigate the relationship between peer assessments of surgeon technical skills and variability in risk-adjusted patient adverse events; (2) investigate the relationship between peer assessments of intraoperative team-based nontechnical practices and variability in risk-adjusted patient adverse events; and (3) use quantitative and qualitative methods to explore the feasibility of using objective, data-driven, computer-based assessments to automate the measurement of important intraoperative determinants of risk-adjusted patient adverse events.

**Results:**

The project has been funded by the National Heart, Lung and Blood Institute in 2019 (R01HL146619). Preliminary Institutional Review Board review has been completed at the University of Michigan by the Institutional Review Boards of the University of Michigan Medical School.

**Conclusions:**

We anticipate that this project will substantially increase our ability to assess determinants of variation in complication rates by specifically studying a surgeon’s technical skills and operating room team member nontechnical practices. These findings may provide effective targets for future trials or quality improvement initiatives to enhance the quality and safety of cardiac surgical patient care.

**International Registered Report Identifier (IRRID):**

PRR1-10.2196/22536

## Introduction

### The Epidemiology of Cardiac Surgery

**Figure 1:**
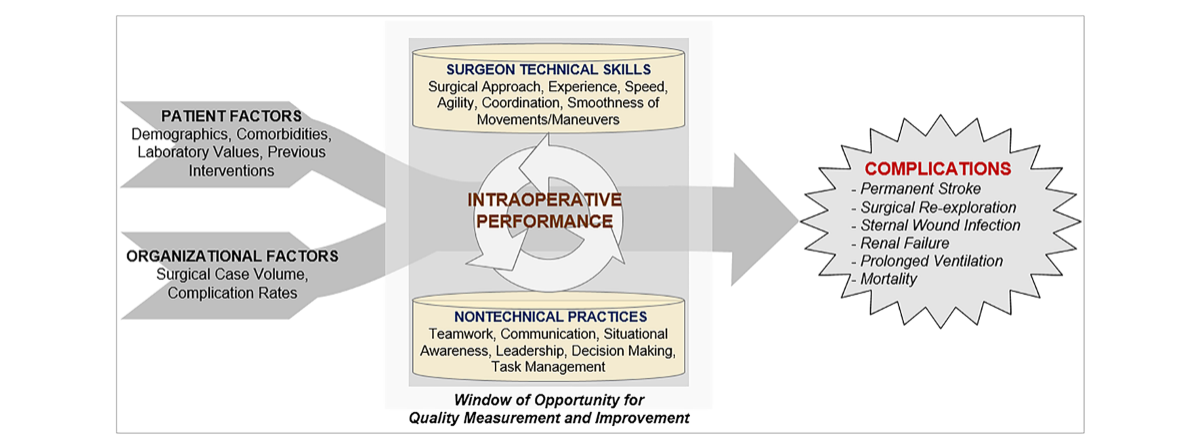
Conceptual model.

Nearly 150,000 coronary artery bypass grafting (CABG) procedures are performed annually in the United States, and it is a procedure associated with a high rate of major adverse events (35% of patients) that vary by hospital [1]. These adverse events increase a patient’s risk of mortality 4.7 fold and are associated with more than US $50,000 in additional health care expenditure per patient [1-4]. While understudied, intraoperative performance (including the surgeon’s technical skills and team-based nontechnical practices) is an important potentially modifiable determinant of operative adverse events (Figure 1) [5,6].

### The Role of Technical Skills in Surgical Outcomes

Prior research has evaluated the association between technical skills (defined as “psychomotor action or related mental faculty acquired through practice and learning pertaining to a particular craft or profession” [7]) and operative outcomes [8]. While taxonomies exist to objectively and reliably assess a surgeon’s technical skills, they are often applied within simulated structured scenarios that may not mimic real-world patient care (Table 1). In one exception, investigators applied the Objective Structured Assessment of Technical Skill (OSATS) [9] to real operative settings including 10 clinician experts who rated a single 25 to 50-minute video segment of a laparoscopic operation from 20 surgeons [5]. Assessments, linked to data from the last 2 years of each surgeon’s experience, were significantly inversely associated with the surgeon’s adverse events and mortality outcomes. In another study, surgical skills were associated with outcomes from cancer surgery [10].

**Table 1:** Application of the Objective Structured Assessment of Technical Skills (OSATS) to cardiac surgery.

Domains	Description	Illustrative high-quality cardiac surgical tasks
Respect for tissue	Gentle tissue handling that does not result in tissue injury	Passing a needle through a coronary artery without tearing the tissue
Time and motion	Economy of motion and maximum efficiency	Efficiency of movement for suturing proximal anastomoses
Instrument handling	Fluid use of instruments and absence of awkwardness	Fluidity of motion between the scrub nurse and surgeon (and back)
Flow of operation	Smooth transitions from one part of the operation to another	Smooth transitions from cannulation (venous and aortic) to anastomosis phase
Suture handling	Efficient knot tying using fluid motions of the hands and fingers	Tying an 8-0 or a 7-0 suture (“microsuture”) resulting in secure knots without causing tissue injury
Steadiness	Absence of tremor motions of the hands	Fine motor movement: passing a needle through a coronary artery without tearing the tissue

### The Role of Nontechnical Practices in Surgical Outcomes

Nontechnical practices (“the cognitive, social, and personal resource skills that complement technical skills, and contribute to safe and efficient task performance” [11]) are both individual and team based. While improvement in these practices has been associated with decreases in operative mortality [12], investigations thus far have focused on developing robust validated taxonomies of behavior with corresponding assessment tools customized to the individual team members’ intraoperative care role. Dominant taxonomies [13-15] include Non-Technical Skills for Surgeons (NOTSS), Anesthetists’ Non-Technical Skills (ANTS), Perfusionists Intraoperative Non-Technical Skills (PINTS), and Scrub Practitioners’ List of Intraoperative Non-Technical Skills (SPLINTS). These taxonomies enable assessments of the following four important categories of nontechnical practices: situation awareness, decision making, communication and teamwork, and leadership/task management. Situation awareness [16] is the process of developing and maintaining a dynamic awareness of the operative situation based on gathering and interpreting data from the operative environment. This domain is essential for effective decision-making [17], representing skills for diagnosing a given situation to inform a judgment about appropriate actions. Successful surgery also depends on social skills allowing multiple individuals with task interdependencies and shared goals to communicate and work effectively as a team [18]. Dysfunctional team dynamics, ineffective communication, and ambiguous leadership [19] account for a substantial proportion of operative adverse events [20].

A surgeon’s nontechnical practices, manifesting as diagnostic failure [21] or a breakdown in teamwork and information sharing [22], may contribute to a higher risk of a major adverse event or death. The largest operative study of NOTSS conducted thus far involved 715 surgical procedures and 11,440 assessments [23]. Surgeons’ nontechnical skills were rated as good (score of 4) in 18.8% of responses, acceptable (score of 3) in 49.1%, marginal (score of 2) in 21.9%, and poor (score of 1) in 0.9%. In a video-based study including 82 cardiac surgeons, there was a 129% increased odds (after adjusting for technical skills) of higher patient safety scores with every 1-point increase in the NOTSS score [6].

### Rationale for the Study

This study will evaluate how operative skills and nontechnical practices impact CABG outcomes. Patients undergoing CABG are at risk of harm due in part to the (1) reconstruction of anatomical structures under high magnification, (2) multiple high-risk phase transitions of care between team members (eg, anesthesiologist and perfusionist), and (3) need for communication and teamwork (eg, instrument handoffs) across many team members.

### Innovation

Our proposed study is novel and innovative for three important reasons. To our knowledge, this study will be the first (1) multicenter intraoperative evaluation of both technical skills and operative team nontechnical practices at scale, (2) study to relate evaluation of intraoperative nontechnical practices with important clinical outcomes, and (3) study to apply a video-understanding platform to high fidelity recorded surgical videos to assess feasibility of automated objective assessments of technical skills and nontechnical practices.

## Methods

### All Aims

Preliminary Institutional Review Board (IRB) review has been completed at the University of Michigan by the Institutional Review Boards of the University of Michigan Medical School. This study will include a single IRB to govern research activities conducted across the collaborating hospitals.

#### Study Population

Our population will include adult patients undergoing electively scheduled CABG operations using cardiopulmonary bypass performed by attending surgeons at six hospitals participating in the Multicenter Perioperative Outcomes Group (MPOG) Collaborative, a national physician-led collaborative of academic and community hospitals, and specialty-specific peer assessors. Surgeons who have operated at their hospital for less than 2 years will be excluded.

#### Digital Recording

**Figure 2:**
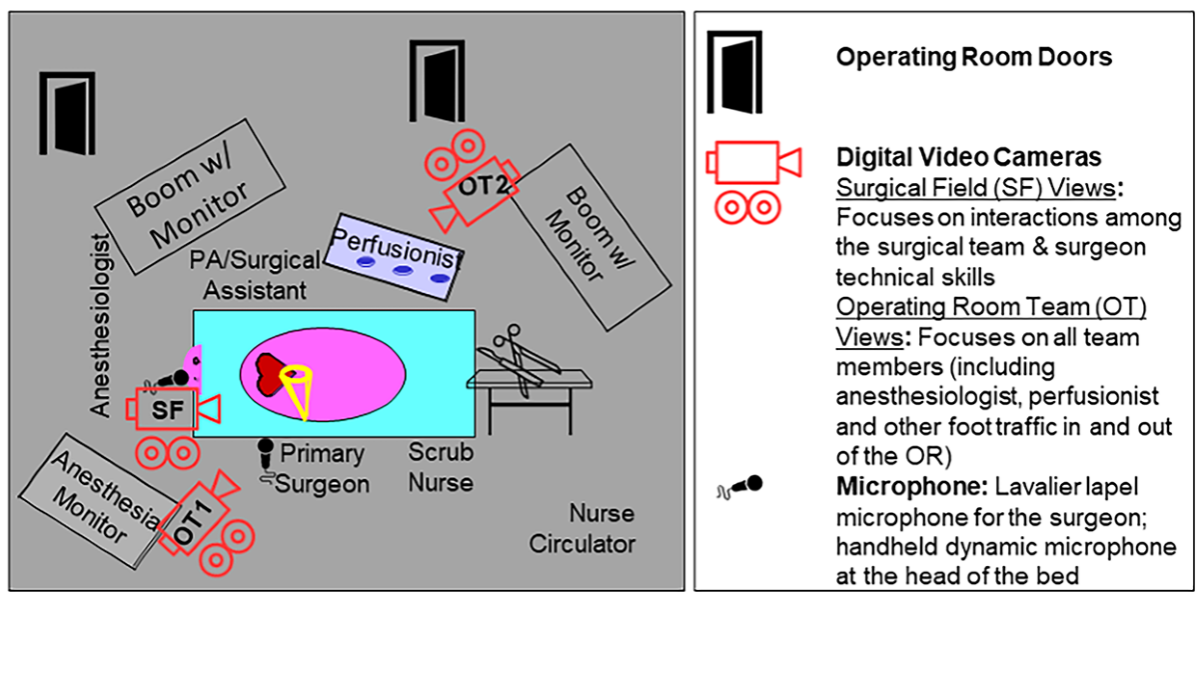
Proposed intraoperative recording configuration. OR: operating room.

We will record 506 CABG operations at six hospitals. The study coordinator will use a randomization protocol from the Data Coordinating Center (DCC) to select, by week, different cardiac surgical operating rooms for video recording. The coordinator will synchronize the cameras with other operating room data sources (eg, intraoperative record as submitted to MPOG) (Figure 2). Three Canon XC15 cameras will be used, with two focusing on operative team members and one focusing on the surgical team. Beyond maximizing nonobstructed visualization, camera positions have been chosen to maximize capture of team member activities and minimize obstruction of existing workflow.

Key transitions in phases of patient care are routinely documented within the intraoperative electronic health record of participating MPOG hospitals. These data are validated and mapped to universal MPOG concepts [24]. Digital recordings will be segmented based upon key transitions in phases of care; operative recordings will begin when the patient enters the operating room and end when the patient exits the operating room.

Study data will be transmitted to the DCC, which will conduct audio and video quality checks across hospitals and recordings. Initially, investigators at the DCC will review the entire recording to fine tune the MPOG event timestamping to the exact second. Given the input operative data, a Hidden Markov Model [25,26] or deep learning–based approach [27] will divide the procedure into temporal segments and associate them with the procedural step labels from the operative script. Standardized segments for assessment will only contain critical operative portions (Table 2).

**Table 2:** Illustrative operative phases for video assessments.

Assessment	Critical portions of the operation	Rationale
Technical skills assessment	Performance of distal arterial and venous anastomoses (exposure is completed → last suture is cut after tying) Performance of proximal arterial anastomosis (initial use of electrocautery to isolate the area for anastomosis → last suture is cut after tying)	Video segment would contain technical skills (eg, economy of motion: creation of anastomosis) that are critical for a successful operation.
Nontechnical practices assessment	Preinduction verification (prior to → end of discussion) Preincision timeout (prior to → end of discussion) Prebypass transesophageal echocardiography (TEE) assessment (surgeon request for TEE → completed discussion between the surgeon and anesthesiologist) Preparation and weaning from bypass (surgeon requests the heart to be filled up → protamine finished)	Video segment would contain nontechnical practices (eg, decision making and communication/teamwork: discussion during the verification and timeout, as well as focusing on ensuring a safe weaning from cardiopulmonary bypass).

#### Hospital Performance Feedback

The DCC will provide monthly reports to hospitals, including number of digitally recorded operations, quality of transmitted digital recordings, and adherence to study operational protocols.

#### Peer Assessment Module

We will use a two-stage process for recruiting candidate assessors as follows: (1) we will poll the Michigan Society of Thoracic and Cardiovascular Surgeons Quality Collaborative (MSTCVS-QC), MPOG, and the Michigan Perfusion Society membership for potential assessors working outside of Michigan or at hospitals not participating in MPOG, and (2) if unable to secure sufficient assessors, we will recruit from the MSTCVS-QC, MPOG, and Michigan Perfusion Society membership.

Assessors, blinded to the hospital and operative team, will provide technical (aim 1) and nontechnical (aim 2) video-based assessments of cardiac surgical operations using a web-based Health Insurance Portability and Accountability Act (HIPAA)-compliant assessment platform.

Each operation will receive at least 12 assessments (three for each provider group), with 20% of assessments rereviewed. Surgeons will assess a surgeon’s (1) technical skills (modified OSATS plus cardiac surgery–specific skills) via a validated 5-point behaviorally anchored scale and (2) nontechnical practices using NOTSS [13]. Anesthesiologists will use ANTS [14], scrub nurses will use SPLINTS [15], and perfusionists will use PINTS for nontechnical assessments (all nontechnical taxonomies will use an 8-point ordinal scale). Segments will capture each operation’s critical phases. Technical skill assessors will be given one operative field camera angle (Cam Surgical Field [SF]) for their assessment (Figure 2). Given the interdependence of intraoperative team members, nontechnical assessors will be provided alternative camera angles depicting the intraoperative team (Cam Operative Team [OT] #1 and #2). Assessors will receive an Amazon coupon for completed reviews.

We will resubmit 20% of edited segments to the same (test-retest reliability) or other assessors (interrater reliability) using an intraclass correlation coefficient α ≥.670 for good concordance [28].

#### e-Learning Training Module

We will use a web-based training and readiness module that will include (1) foundational knowledge of the relevant tool and (2) video examples of correct identification, categorization, and assessment. Assessors will have to reach 70% agreement with gold standard (investigative team) assessments to contribute to the study. A median 70% agreement with reference assessments will serve as a basis for assessor eligibility to conduct real case assessments [29].

#### Clinical Complications

We will calculate each surgeon’s adverse event rate for the previous 2 years using each hospital’s Society of Thoracic Surgeons (STS) data.

### Aim 1: Investigate the Relationship Between Peer-Rater Assessments of a Surgeon’s Technical Skills and Variability in Risk-Adjusted Patient Adverse Events

#### Approach

We will conduct peer-reviewer assessments of recorded CABG operations at six MPOG hospitals (representing 36 surgeons) to associate technical skills with major adverse events.

Each surgical operation will be divided (using our video segmentation protocol) into prespecified phases containing the most critical operative portions. The DCC will distribute video segments for surgeon assessment via our HIPAA-compliant assessment platform that will provide assessors with a view of the operative field (from Cam SF, Figure 2). Surgeon assessors will provide domain-specific and overall summary judgements (using a modified OSATS taxonomy). Twenty percent of segments will be resubmitted for review to test assessor reliability. We will associate the average assessments with the adjusted risk of major morbidity and mortality over the prior 2 years for each surgeon.

#### Measures

Our primary exposure will be the average summary assessment of each surgeon’s technical skills. The primary outcome will be a surgeon’s STS composite major morbidity or mortality (ie, permanent stroke, surgical re-exploration, deep sternal wound infection, renal failure, prolonged ventilation, or operative mortality) rate. We will use clinical data from each center to adjust for covariates incorporated within the STS risk prediction models [30,31].

#### Analytical Plan

We will use linear mixed effect models to model assessments of surgical procedures where assessors and surgeons are included as random effects. We will quantify variation in peer-assessor assessments of a surgeon’s technical skills and use the intraclass correlation coefficient to measure interassessor reliability. We will use predictions of each surgeon’s technical skills from the linear mixed effect models as summary measures of a surgeon’s technical skills in subsequent analyses. Generalized linear mixed effect models with a logit link will then be used to associate a surgeon’s technical skills with our composite outcome. We will model surgeons and hospitals as random effects, accounting for the nesting structure of the data (ie, patients nested within surgeons and hospitals). We will adjust for patient and surgeon factors by including them as fixed effects in the models. The factors of interest are summary measures of a surgeon’s technical skills, which are included as surgeon-level explanatory variables. We will consider the overall assessments of a surgeon’s technical skills, averaged across three assessors and each domain individually.

#### Power Analysis

We use simulations to evaluate statistical power for a two-sided test (α=.05). Our analysis will be based on outcomes for approximately 7200 operations over 2 years from 36 surgeons (approximately 100 operations per surgeon) at six hospitals. We estimate having approximately 98% power in detecting an odds ratio of 0.85 per one unit (standardized) increase in a surgeon’s technical skills for the rate of adverse events.

### Aim 2: Investigate the Relationship Between Peer-Rater Assessments of Intraoperative Team-Based Nontechnical Practices and Variability in Risk-Adjusted Patient Adverse Events

#### Approach

We will leverage each hospital’s intraoperative electronic health record system for video segmentation, using in part precomputed, validated, publicly available MPOG phenotypes [24,32]. Segments will be reviewed by at least 12 assessors (three per provider group). We will assess the association between peer assessments of nontechnical practices and surgeon measures of postoperative major morbidity and mortality, adjusted for patient risk factors and surgeon technical skills.

#### Measures

Our primary exposure will be the average summary peer assessment of each provider’s nontechnical practices. Similar to aim 1, the primary outcome will be the rate of major morbidity or mortality, adjusting for clinical covariates [30,31].

#### Analytical Plan

We will use generalized linear mixed effect models with a logit link to associate peer-assessor assessments of nontechnical practices of the surgeon with the surgeon’s STS composite score for major morbidity and mortality. Models will be similar to those described in aim 1, although we will include average summary measures of surgeon’s nontechnical skills as surgeon-level explanatory variables and hospital-level average summary measures of anesthesiologists, perfusionists, and scrub nurses. Both overall summary measures and individual scale domains will be considered. We will focus primarily on assessing the effects of nontechnical practices on morbidity and mortality rates, while adjusting for patient-level risk factors and a surgeon’s technical skills. We will explore the influence of nontechnical practices on the relationship between a surgeon’s technical skills and our composite endpoint by including nontechnical practices as an interaction term in models with technical skills.

#### Power Analysis

The power analysis is based on approximately 7200 cases across 36 surgeons from six hospitals. As surgeon’s nontechnical practices are considered a surgeon-level variable, there will be sufficient power in detecting the same effect sizes as reported in aim 1.

### Aim 3: Explore the Feasibility of Using Objective, Data-Driven, Computer-Based Assessments to Automate the Identification and Tracking of Significant Intraoperative Determinants of Risk-Adjusted Patient Adverse Events

High-dimensional computer-based assessments of digital recordings will be used to recognize and track human activity (computer vision). Computer vision focuses on training computers to derive meaning from visual imagery. Video understanding, a specialty within computer vision, focuses on identifying and tracking objects over time from video and developing mathematical models to train computers to extract the meaning within these moving images. This field may offer unparalleled capabilities for conducting objective peer assessments by automatically identifying and tracking human activity comparable to that of expert human assessors.

#### Background

##### Surgical Technical Skills

Video understanding may address some of the limitations in traditional mentored or simulation-based approaches for assessing a surgeon’s technical skills, including human assessor bias and limited scalability. Prior investigations have documented the reliability of video-based surgical motion analyses for assessing laparoscopic performance as compared to the traditional time-intensive human assessor approach [33,34]. Azari et al compared expert surgeon’s rating assessments to computer-based assessments of technical skills [35]. Computer-based assessments had less variance relative to expert assessors. Sarikaya et al evaluated the feasibility of computer-based methods for technical skill assessment involving 10 surgeons having varying experience with robotic-assisted surgery [36]. This evaluation included acquiring 99 unique videos with 22,467 total frames and the development of a state-of-the-art deep learning–based surgical tool tracking system. The quantitative assessment against gold standard (human annotated) tool tracks found a 90.7% mean average precision over all test videos across all surgeon skill levels.

##### Nontechnical Practices

Nontechnical practice assessments have predominantly occurred within simulated environments and relied on trained human observers [37,38]. Investigators have not evaluated whether video understanding could provide an objective alternative for high-fidelity assessments of nontechnical practices in real-world operative environments, generalizable across hospitals with varied operating room layouts and camera configurations. Video understanding may be used to assess features aligned with nontechnical practices without relying on verbal communication [39]. Video understanding requires time-limited human observer involvement to provide labels for training algorithms after which the automated system may be deployed at scale.

#### Approach

We will explore the feasibility of using a video-understanding platform to identify important features associated with assessor ratings in recorded operations. To support developing the video-understanding platform, we will conduct interviews and site visits at a subset of low- and high-performing hospitals to enhance the understanding of a hospital’s contextual characteristics (eg, culture) and important “usual practices.”

##### Video Understanding

The video-understanding approach will focus on two specific techniques (ie, visual detection and visual tracking), which will be applied to identify and measure surgeons’ technical skills (aim 1) and team-based nontechnical practices (aim 2). We will apply ambiguity reduction across the three time-synchronized video recordings to harmonize (rather than duplicate) *elements* within and across video angles. We will use proven methods for video understanding (eg, boosting [40] and deep learning [41]). We will use boosting for cases of limited data and deep learning for cases of ample data. We will learn detection models to ascertain kinematic features potentially associated with surgical technical skills (eg, path length of the surgeon’s suturing and nonsuturing hands) and nontechnical practices (eg, identifying and tracking the gaze direction of team members at critical times of the surgical procedure) based on aims 1 and 2. We will learn these features using the following mutually exclusive data sets containing video segments: (1) training data set (used for training the video-understanding algorithms); (2) computer vision validation data set (used to mitigate risk of overfitting [eg, the video-understanding algorithms]); (3) computer vision testing data set (used for computing the error statistics of the computer vision system to meet human feature annotation); and (4) study set (video segments for peer assessments). Investigators will observe the raw video from the training data set to provide bounding-box annotations for each feature, within contextual feedback provided by members of the investigative team who work in the operating room. A certain detection model is initialized with a random set of parameters, and then, the training algorithm iteratively refines them based on the model’s empirical performance (ability to automatically detect the phenomena bounding boxes) based on the annotations in the training data. The validation set is used during this training process to protect against overtraining and bias. Some technical assessments will require detection in a video frame and tracking of the detected object throughout the video frames (“visual tracking”). For example, to measure the surgeon’s economy of motion, we will detect the surgeon’s hands at frame t, track the surgeon’s hands at all future frames t+k, and then compute a trajectory of the centroid of the detected bounding boxes. We will use both classical physics-based tracking models (eg, Lucas-Kanade tracking [42]) and modern deep learning–based methods [43]. We will compute a range of validated kinematic features [35] and quantifiers of economy of motion (eg, path length of the surgeon’s suturing and nonsuturing hands, and variance of local change in the trajectory against a linear or smoothed trajectory).

##### Qualitative Interviews

Concurrent with developing and testing the video-understanding platform, we will randomly select up to four of the six hospitals (equal representation of low- and high-outlier hospitals) participating in aim 2 for more detailed investigation. We will conduct semistructured interviews with interdisciplinary cardiac surgery operating room team members. To enhance our understanding of technical and nontechnical operating room practices, we will collect data (through interviews with intraoperative team members) concurrent with conducting analyses. We will develop a semistructured interview guide to encourage new and/or unexpected ideas or concepts to surface. For each interview, the interviewer will play back video segments from an operation involving the interviewee and ask the interviewee to describe his/her role within that operative phase. The interviewer will ask questions seeking to better understand team member roles and influences on technical skills and nontechnical practices. We expect the guide will consist of seven to nine open-ended questions with probes. Interviewers will participate in a 3-day training program at the University of Michigan Health Communications Laboratory. Interviews will continue until reaching informational redundancy “saturation” at each hospital. We will (1) conduct 40 to 60-minute interviews in private rooms, (2) digitally record and transcribe transcripts verbatim, (3) compare 10% of transcripts (and correct as needed) against the recordings, and (4) provide interviewees with a gift certificate. We expect that (1) in reviewing the videos, providers will complement peer assessments regarding how and why contextual factors influence performance (technical and nontechnical) and (2) interviewees will validate the video content to maximize our video understanding algorithm’s fidelity. Thus, our interview findings will improve our interpretation of the video content to iteratively inform and enhance our video-understanding platform’s training.

#### Measures

Our primary outcome will be the features derived from the video-understanding platform, which will be compared to a gold standard human identifying the same features. Features, as economy of motion, are derived from the raw output of the video-understanding platform, which naturally performs visual detection and visual tracking. The gold standard uses the analogous “raw output” from humans and the same method for the computation of the derived feature.

#### Analytical Plan

We will assess our video-understanding platform’s ability to correctly identify and track features within our testing data set. Using the raw video in the testing set, we will provide the necessary bounding-box annotations for each feature, which will be compared to the automatically generated features from the video-understanding system using standard metrics (eg, intersection over union [44] and DICE coefficient [45]). For example, when we compute the economy of motion of the surgeon’s hand, we will provide bounding-box annotations of the surgeon’s hand. The video-understanding system will use these annotations to learn a mathematical visual detection model capable of producing the detections of the hand on novel video. Thereafter, the economy of motion feature will be derived on the output bounding boxes. We plan a two-phased analysis. First, we will measure agreement and associate each feature with each component of the technical and nontechnical assessments (specific to each operative phase) using Pearson/Spearman correlation coefficients or Kendall tau, depending on data distribution. Second, we will identify the best combination of video-understanding features that are most closely associated with technical and nontechnical score domains (specific to each operative phase). We will use regression (eg, linear, ridge, and deep) to model each domain and technical and nontechnical summary scores as dependent variables, including features from the video-understanding platform as independent variables. We will (1) select features using variable selection and (2) quantify the magnitude of information in peer assessment that can be identified by the computer using generalized R squares.

## Results

**Figure 3:**
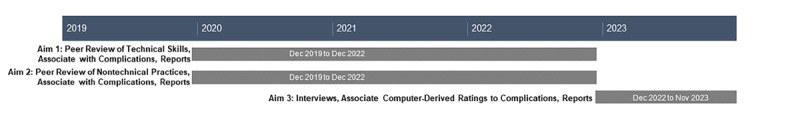
Study timeline.

The project has been funded by the National Heart, Lung and Blood Institute in 2019 (R01HL146619). Results of aims 1 and 2 will likely yield assessments that identify a wide range of variations in both surgeons’ technical skills and nontechnical practices as has already been documented in the literature. Where our study will make an important contribution is in associating these assessments with adverse event rates. The novel contribution of aim 3 will be to associate computer-based assessments with adverse event rates, as a more objective and reliable replacement for human peer assessors, moving us closer to our overall goal of improving outcomes for cardiac surgery patients. We will use the study results to develop data-driven technical skills and nontechnical practice coaching interventions across a subset of hospitals. We plan to undertake our study over a 5-year period (Figure 3).

## Discussion

### Strengths

There is increasing demand from the public and payers to improve health care value (quality divided by expenditures). Despite wide variability in cardiac surgical quality and robust clinical data from the STS for risk adjustment and outcomes ascertainment, only 2% of hospital variability in some outcomes are explainable by currently recorded data elements [46]. Analysis of operative videos may reveal unique opportunities for advancing operative quality improvement beyond that provided through traditional data sources [47].

Our proposed study, leveraging the infrastructure and track record of two established physician-led quality collaboratives integrated with a cutting-edge scalable video-understanding platform, will advance our understanding of how surgical skills and nontechnical practices impact outcomes. Our approach aimed at identifying key modifiable intraoperative determinants of major adverse events may likely be applied to approximately 200,000 additional cardiac surgical procedures involving valve repair or replacement, aortic procedures, and percutaneous cardiac procedures (eg, transcatheter aortic valve replacement) or other high-risk noncardiac surgical specialties (eg, neurosurgery, orthopedics, and head and neck reconstructive surgery).

### Limitations

Although unlikely, there are a few potential challenges with this study.

#### Aims 1 and 2

There is a remote possibility that we will not find that the investigated technical skills are associated with adverse events. If needed, we will expand our review of surgical operations to include (1) hospitals with lower operative volume, (2) longer segments for peer rating, (3) an expanded list of operative phases that might distinguish between high- and low-performing surgeons, and (4) high-risk or technically challenging operations. We will consider expanding to other hospitals if (1) hospital variability in adjusted adverse events is less than anticipated or (2) we are unable to amass sufficient digital recordings from our initial six hospitals.

If needed, we will (1) expand our sampling pool of assessors to include providers who have expressed desire to partner on this project but were not selected initially, (2) provide monthly feedback and engagement support to participating assessors, and (3) provide expanded assessor training and calibration.

#### Aim 3

Our video-understanding platform may not be completely automated. Alternatively, we will consider a semiautomated platform that relies, for instance, on a human periodically manually annotating the relevant features in the video at a certain segment and then allowing the video-understanding platform to interpolate those annotations.

## Abbreviations

ANTS: Anesthetists’ Non-Technical Skills
CABG: coronary artery bypass grafting
DCC: Data Coordinating Center
HIPAA: Health Insurance Portability and Accountability Act
MPOG: Multicenter Perioperative Outcomes Group
MSTCVS-QC: Michigan Society of Thoracic and Cardiovascular Surgeons Quality Collaborative
NOTSS: Non-Technical Skills for Surgeons
OSATS: Objective Structured Assessment of Technical Skill
OT: operative team
PINTS: Perfusionists Intraoperative Non-Technical Skills
SF: surgical field
SPLINTS: Scrub Practitioners’ List of Intraoperative Non-Technical Skills
STS: Society of Thoracic Surgeons
